# Comparative Analysis of Pigments, Phenolics, and Antioxidant Activity of Chinese Jujube (*Ziziphus jujuba* Mill.) during Fruit Development

**DOI:** 10.3390/molecules23081917

**Published:** 2018-08-01

**Authors:** Qianqian Shi, Zhong Zhang, Juanjuan Su, Jun Zhou, Xingang Li

**Affiliations:** 1 College of Forestry, Northwest A&F University, Yangling 712100, China; Shiqq@nwsuaf.edu.cn (Q.S.); Zhangzhong@nwsuaf.edu.cn (Z.Z.); 18792845316@163.com (J.S.); 2 Key Comprehensive Laboratory of Forestry of Shaanxi Province, Northwest A&F University, Yangling 712100, China; 3 School of Biological Science and Engineering, North Minzu University, Yinchuan 750021, China; zhoujunbo@163.com; 4 Research Center for Jujube Engineering and Technology of State Forestry Administration, Northwest A&F University, Yangling 712100, China

**Keywords:** Chinese jujube (*Ziziphus jujuba* Mill.), anthocyanin, carotenoid, phenolic compounds, antioxidant activity

## Abstract

Chinese jujube (*Ziziphus jujuba* Mill.) fruit are much admired for their unique flavor and high nutritional value. This study tracks changes in skin color and antioxidant activity over six stages (S1 … S6) of fruit development in two cultivars of jujube, ‘Junzao’ and the color mutant ‘Tailihong’. The study records the changing levels of chlorophylls, carotenoids, anthocyanins, and phenolic compounds during fruit development. Contents of chlorophylls, β-carotenes and anthocyanins decreased throughout the developmental stages in two jujube cultivars, while lutein contents decreased at first and then increased to a maximum at S6. The levels of total phenolics, total flavonoids, total flavanols, total anthocyanins, procyanidin B1, procyanidin B2, procyanidin B3, (+)-catechin, quercetin, and ferulic acid are significantly higher in ‘Tailihong’ than in ‘Junzao’ before the onset ripening (before S3). However, after S3 the level differences of these components in the two cultivars are not significant. In both cultivars, antioxidant activity reduces gradually throughout fruit development. Our findings indicate how the skin color of jujube fruit during maturation is due to changes in the levels of flavonoids, carotenoids, and anthocyanins. The color changes are also associated with changes in antioxidant activity.

## 1. Introduction

Chinese jujube (*Ziziphus jujuba* Mill.) is indigenous to China and belongs to the Rhamnaceae. This fruit crop has been domesticated for more than 7000 years in China [1], where about 6.25 million tons of dry fruit are produced annually from a cultivated area of approximately two million hectares [2]. The jujube is well known as a traditional medicine for its numerous pharmacological activities [3,4,5] and is also much admired for its unique flavor and high nutritional value. Recent studies have revealed that jujube fruits contain various functional compounds such as vitamin C, amino acids, triterpene acids, polysaccharides, and polyphenols [6,7].

Polyphenols are one of the most important classes of secondary metabolites in plants and are associated with strong antioxidant activity [8,9]. Polyphenols include phenolic acids, flavonoids, flavanols, and anthocyanins which are closely related to the intensity of fruit skin color [10,11,12]. Anthocyanins are important water-soluble pigments and these are responsible for the red colors of many fruits, including apples, grapes, strawberries, and litchi [13,14,15]. In addition, carotenoids also contribute to the formation of the red color of red peppers [16] and carrots [17]. However, studies of the coloration in jujube are limited [18,19], although a number of studies have reported the antioxidant activities of phenolic compounds [20]. The types of anthocyanin present and the contents of chlorophyll and carotenoid have also not been reported.

To investigate pigment formation in Chinese jujube fruit during development and ripening, we selected two jujube cultivars. The first was the commercial cultivar ‘Junzao’ whose skin color changes from green to white during early ripening and later turns red. The second was a color mutant ‘Tailihong’, in which the skin color changes from purple-red in young fruit to yellow at the beginning of ripening and then to red at full ripening. Variations in the levels of chlorophylls, carotenoids, anthocyanins phenolic compounds and antioxidant activity were determined. Understanding the pigment changes of jujube fruit during ripening is important since it does allow a better understanding of the main pigments responsible for the color of ripping fruit and the properties of the fruit.

## 2. Results and Discussion

### 2.1. Skin Color Changes during Fruit Development and Ripening in ‘Tailihong’ and ‘Junzao’

**Figure 1:**
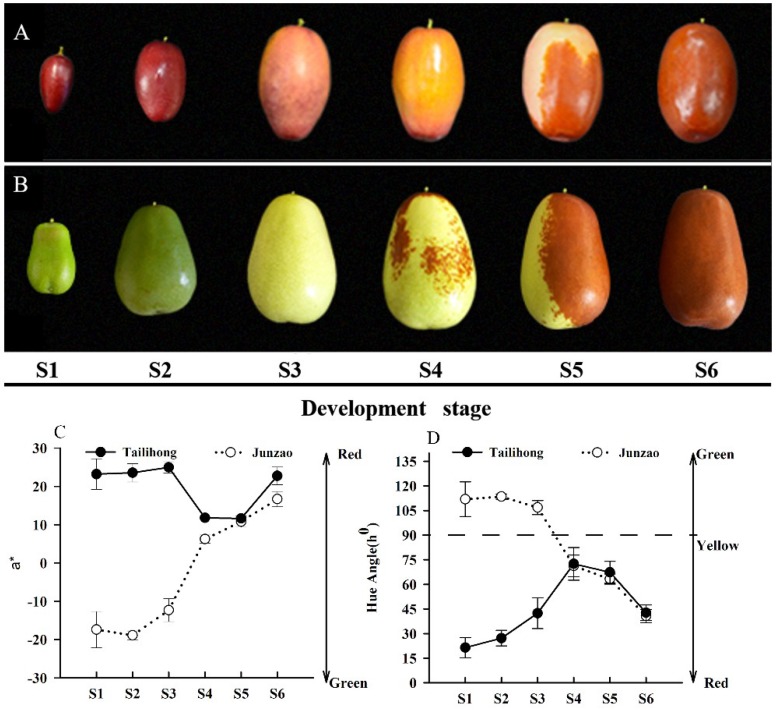
Color changes of jujube fruit development. Photographs of the six development stages S1–S6 of (**A**) ‘Tailihong’; (**B**) ‘Junzao’; (**C**) Fruit development color a*; (**D**) Fruit development color h^0^. Developmental stages S1–S6 correspond to days 30, 50, 80, 90, 100, and 110 after anthesis.

During fruit development (Figure 1A,B), the pericarps of the two cultivars followed different patterns of color change (a* and h^0^). The ‘Tailihong’ fruit had a hue angle, h^0^ < 30, which appears as red-purple at S1 (immature) (Figure 1D). The ‘Junzao’ fruit were different, showing a rapid decrease in hue angle starting at S4 (reddish, mature). Over about 20 d, the hue angle fell from 71.24 to 40.68°, which corresponds to changes in appearance from bright green to red (Figure 1C). The pericarp color of ‘Tailihong’ showed a declining trend for hue angle from S4 to S6 (red, the full maturity stage), reaching a constant value of 42°. In contrast, the values of the parameter a* of ‘Junzao’ were positive and increased linearly from S4 to S6. The a* values of ‘Tailihong’ were lower in S4 (yellow, mature) and S5 (half red, mature). These values are consistent with the change in fruit pericarp color from light yellow to red (Figure 1D). Although the two cultivars showed differences in their coloration during development, the final color parameter values were similar to those reported previously for jujubes [21]. 

### 2.2. Pigment Components in the Skins of ‘Tailihong’ and ‘Junzao’

**Figure 2:**
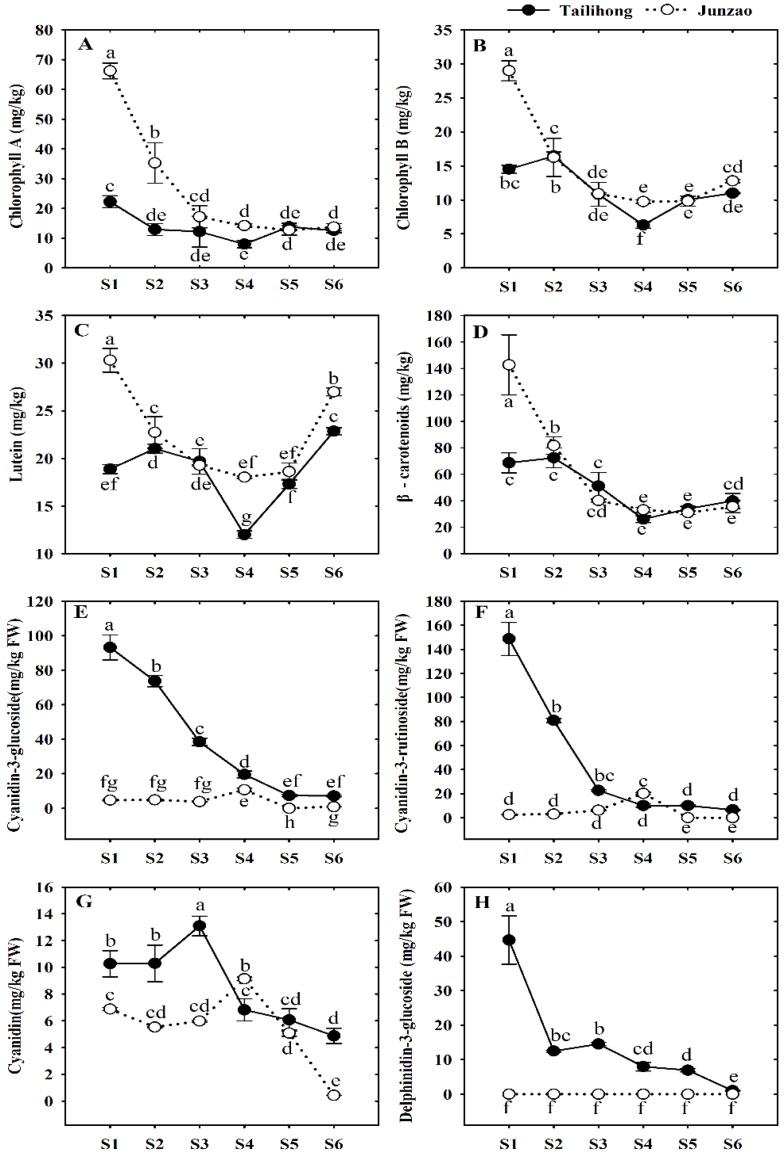
Pigment changes of the skins of the jujube cultivars ‘Tailihong’ and ‘Junzao’. (**A**–**D**) Contents of chlorophyll a, chlorophyll b, lutein, and β-carotene; and (**E**–**H**) contents of cyanidin-3-glucoside, cyanidin-3-rutinoside, cyanidin, and delphinidin-3-glucoside. Different letters (a–h) indicate significant differences at *p* < 0.05 by Duncan’s test. Developmental stages S1–S6 correspond to days 30, 50, 80, 90, 100, and 110 after anthesis.

Chlorophyll, carotenoid, and anthocyanin are the most important color pigments in fruit [22]. The content of chlorophyll a is maximal at S1 (22.24 mg/kg FW, 66.19 mg/kg FW) in ‘Tailihong’ and ‘Junzao’, while the content of chlorophyll b in ‘Tailihong’ showed a peak at S2 (16.46 mg/kg FW). During the development and ripening processes, the contents of chlorophylls a and b in the two jujube cultivars decreased until full maturity at S6. The chlorophyll a content of ‘Junzao’ was markedly higher than that of ‘Tailihong’ before S5 (half red, mature) but not significantly different at the other stages (Figure 2A). The changes in chlorophyll b were consistent with this pattern (Figure 2B). This change pattern indicates chlorophylls play an important role in determining the fruit’s green color in the initial stages in ‘Junzao’. This observation is consistent with those described previously by Alniami et al. [23], who found the content of chlorophyll decreased steadily during the whole period of fruit ripening in jujube. The lutein content of the two jujube cultivars decreased sharply before S4 but later increased (Figure 2C). It is noteworthy that the content of β-carotene decreased throughout development (Figure 2D). This change pattern indicates lutein seem to be associated with the formation of the color in jujube fruit at the full maturity stage (S6). 

Anthocyanidin components, cyanidin-3 glucoside, cyanidin-3-rutinoside, cyanidin, and delphenidin-3-glucoside in the two jujube cultivars, were all identified and confirmed by high-performance liquid chromatography (HPLC) retention times, PhotoDiode Arraray (PDA) and LC-ESI-MS/MS (seen in Figures S1 and S2 of Supplementary Information). However, the delphenidin-3-glucoside was detected only in ‘Tailihong’. The contents of cyanidin-3 glucoside peaked at S1, and then gradually decreased in ‘Tailihong’; levels were higher than in ‘Junzao’ during the whole ripening period (Figure 2E). Similar change patterns were found for the contents of cyanidin-3-rutinoside, cyanidin and delphenidin-3-glucoside (Figure 2F–H). These findings are similar to this of Bastos et al. [24], who show that the content of anthocyanin increased at the beginning of the Indian jujube fruit coloration period and decreased until full maturity. This change pattern indicates anthocyanin is the primary pigment responsible for the formation of the red color before ripening onset (before S3) in ‘Talihong’.

These results show the higher levels of β-carotene, lutein, and anthocyanin contribute to the redness color of ‘Tailihong’ before the fruit ripens, meanwhile, the contents of the chlorophyll that decreased during this process of ‘Junzao’ contribute to the color change from green to white-like; and the similar red color of jujube fruit at maturity (S6) is due to a blend of various amounts of lutein and possibly some trace amounts of anthocyanins.

### 2.3. Contents of Individual Phenolic in the Skins of ‘Tailihong’ and ‘Junzao’

Phenolics in fruit are important to consumers for their antioxidant activity [25]. Nineteen phenolic compounds, including five flavanols (procyanidin B1, procyanidin B2, procyanidin B3, (−)-epicatechin and (+)-catechin), six phenolic acids (gallic acid, chlorogenic acid, caffeic acid, ferulic acid and cinnamic acid) and five flavonols (quercetin-3-galactoside, quercitrin-3-glucoside, quercetin-3-rutinose, quercetin-3-rhamnoside and quercetin) were well separated and quantified by HPLC. Similar types of phenolic compounds have been described previously in jujubes [11,18,26,27]. 

**Figure 3:**
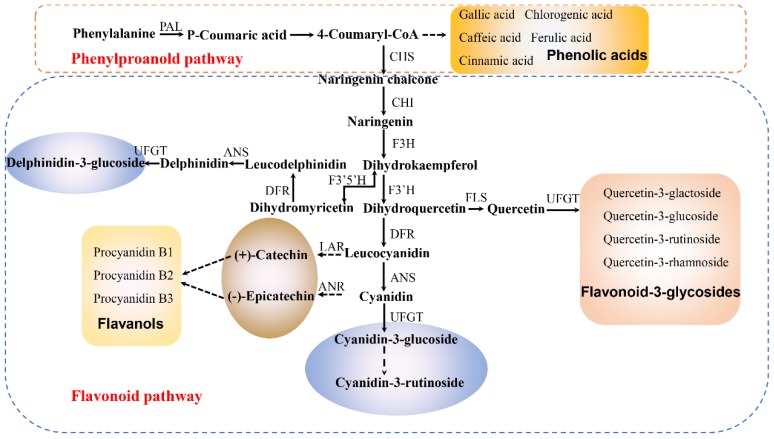
The flavonoid metabolic pathway of the jujube cultivars ‘Tailihong’ and ‘Junzao’. Enzyme names are abbreviated as follows: phenylalanine ammonia-lyase (PAL); chalcone synthase (CHS); chalcone isomerase (CHI); flavanone 3-hydroxylase (F3H); flavonoid 3′-hydroxylase (F3′H); flavonoid 3′,5′-hydroxylase (F3′5′H) dihydroflavonol4-reductase (DFR); anthocyanidin synthase (ANS); flavonol synthase (FLS); leucoanthocyanidin reductase (LAR); anthocyanidin reductase (ANR); UDP glycose: flavonoid 3-*O*-glucosyl transferase (UFGT). Figure based on reference [29].

Flavanols are the products of the flavonoid metabolic pathway (Figure 3). (+)-Catechin and (−)-epicatechin contribute to the formation of procyanidins. Three monomers ((−)-epicatechin, (+)-catechin and quercetin) and three dimers (procyanidins B1, B2 and B3) were identified and quantified (Table 1). With fruit development, concentrations of procyanidin B1, procyanidin B2, procyanidin B3, and (+)-catechin increased gradually in both cultivars during early developmental and then decreased to maturity. The levels of three types of procyanidin, (−)-epicatechin and (+)-catechin were higher in ‘Tailihong’ than in ‘Junzao’. The procyanidin contents may lead to high antioxidant activity and explain the bitter taste in fruit [28]. 

The levels of nearly all the phenolic acids decreased with the development in both cultivars. Gallic acids were at higher levels of the whole phenolic acid derivatives levels in the two jujube cultivars. It varies from ‘Tailihong’ from S1 (61.22 mg/kg FW) to S4 (83.87 mg/kg FW). Similar results have been reported previously for jujube [18]. The levels of chlorogenic acid decreased from 94.75 ± 3.25 to 21.19 ± 4.46 mg/kg FW and 73.67 ± 4.77 to 19.43 ± 0.01 mg/kg FW in ‘Tailihong’ and ‘Junzao’, respectively (Table 1). While the changes we observed are similar to those reported by Wang et al. [11] the levels we report are more than two-fold higher.

Quercetin glycosides are the main flavonols found. In ‘Tailihong’, the concentrations of quercetin-3-glucoside and quercetin-3-rhamnoside, except quercetin-3-rutinose, began to rise before S3 (29.27–155.1 mg/kg FW, 30.20–69.89 mg/kg FW) and then decreased to S6 (9.5 mg/kg FW, 24.79 mg/kg FW). The contents of the four flavonols decreased continuously during fruit development in ‘Junzao’ and were at higher levels than in ‘Tailihong’ (Table 2).

### 2.4. Total Content of Phenolics, Flavonoids, Flavanols, and Anthocyanins in the Skins of ‘Tailihong’ and ‘Junzao’

**Figure 4:**
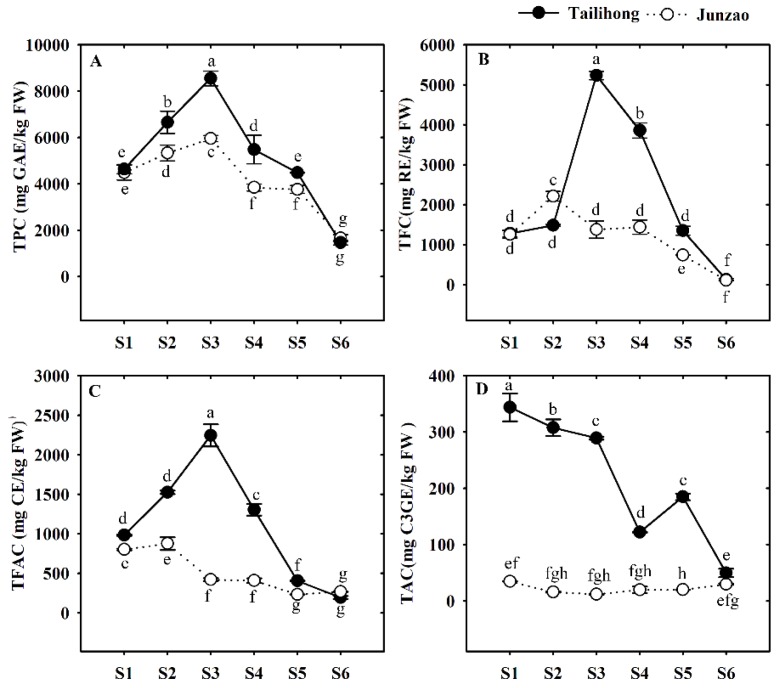
Total phenolics (TPC), total flavonoids (TFC), total flavanols (TFAC), and total anthocyanins contents (TAC) in the fruit skins of the jujube cultivars ‘Tailihong’ and ‘Junzao’ during development. (**A**) TPC in mg GAE/kg FW; (**B**) TFC in mg RE/kg FW; (**C**) TFAC in mg CE/kg FW; and (**D**) TAC in mg C3GE/kg FW. Abbreviations: GAE, gallic acid equivalent; RE, rutin equivalent, CE, catechin equivalent, and C3GE, cyanidin 3-gucoside. Different letters (a–h) indicate significant difference at *p* < 0.05 by Duncan’s test. Developmental stages S1–S6 correspond to days 30, 50, 80, 90, 100 and 110 after anthesis.

Total phenolics, flavonoids, flavanols, and anthocyanins in the skins of the two jujube cultivars were measured during the six developmental stages (Figure 4). The total phenolics content (TPC) values peaked at S3 (8550.81 mg GAE/kg FW, 5952.83 mg GAE/kg FW) but then decreased strongly till S6 (1465.23 mg GAE/kg FW, 1661.41 mg GAE/kg FW). This observation is similar with that of Gao et al. [30], who found levels of TPC of 5418 mg GAE/kg FW. Meanwhile, The TPC contents were higher in ‘Tailihong’ than in ‘Junzao’ throughout fruit development, except at S6 (red, fully mature) (Figure 4A). Interestingly, the levels of TPC in ‘Tailihong’ in this study were similar to that of ‘pear-jujube’ (769.97 mg GAE/ 100 g FW) observed by Wu et al. (2012) [12]. 

The total flavonoids content (TFC) values differed in the two cultivars with a fruit development stage (Figure 4B). The contents of TFC rose to a maximum at S3 (5232.85 mg RE/kg FW) before declining toward commercial maturity (S6) (129.2 mg RE/kg FW) in ‘Tailihong’. However, in ‘Junzao’, TFC peaked at S2 (2209.19 mg RE/kg FW) and then gradually decreased. At all stages, the TFC levels were almost two-fold higher in ‘Tailihong’ than in ‘Junzao’. The TFC contents showed similar trend variations to TPC. 

Total favanols content (TFAC) showed similar developmental trends as TPC and TFC in the two cultivars (Figure 4A–C). Although TFAC levels in ‘Tailihong’ were initially higher than in ‘Junzao’, both exhibited very similar change patterns during ripening (Figure 4C). 

In both jujube cultivars, TPC, TFC, and TFAC increased slightly in the early stages of development, followed by decreases until S6 (red, fully mature). The pattern of change was similar to that reported by Zozio et al. [31], who found that the levels of TPC increased initially and then decreased during ripening. These results indicate the phenolics levels are related not only to the maturity stage but are also associated with the red color of jujube fruit at maturity, which was consistent with the reported by Xie et al. [18]. Interestingly, the content of TPC in jujube fruits was higher than other common fruits, such as apple, red grape, and cherries [32].

In fruits and vegetables, anthocyanins are used widely as indices of the developmental stage. Total anthocyanins content (TAC) decreased continuously throughout development in ‘Tailihong’, being consistently higher than in ‘Junzao’, in which the levels of TAC generally remained low (34.66 to 11.64 mg C3GE/kg FW) (Figure 4D). The TAC values in ‘Tailihong’ ranged from 343.77 to 49.46 mg C3GE/kg FW. These levels are far higher than the reported of cv. Dongzao (15 to 4 mg C3GE/kg FW) by You et al. [33]. This difference may be partly due to genotype and cultivar.

To deeply understand the relationship between phenolics and color difference in jujube skins, correlation analysis was performed among the TPC, TFC, TFAC, TAC, and CIE a* h* parameters in the six developmental stages for each jujube cultivar (seen in Table S1 of Supplementary Information). a* showed a closely related to TPC (ǀrǀ = 0.840, *p* < 0.05) and TFAC (ǀrǀ = 0.879, *p* < 0.05), while h* showed a significant correlation with TPC (r = 0.900, *p* < 0.05), TFC (r = 0.827, *p* < 0.05) and TFAC (r = 0.875, *p* < 0.05) in ‘Junzao’. However, the CIE a* h* values exhibited little correlation (r < 0.600) with polyphenols in ‘Tailihong’ (seen in Table S2 of Supplementary Information). This result may be due to the special coloration during the jujube developmental in ‘Tailihong’. 

Therefore, the composition and content of phenolics could be an indication of co-pigmentation in the skin of jujube fruit, which reflects the maturity level.

### 2.5. Antioxidant Activity in the Skins of ‘Tailihong’ and ‘Junzao’

**Figure 5:**
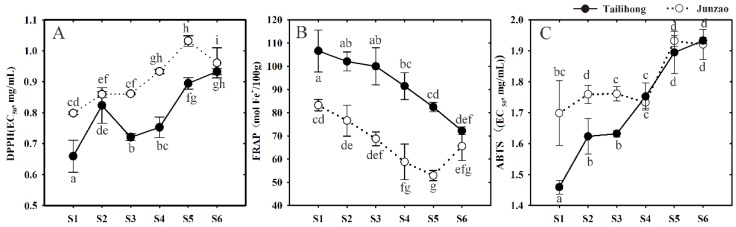
Change of antioxidant activity in the skins of jujube fruit cultivars ‘Tailihong’ and ‘Junzao’ during development. (**A**) DPPH radical scavenging capacity; (**B**) FRAP ferric reducing antioxidant capacity and (**C**) ABTS cation radical scavenging capacity. Different letters (a–g) indicate significant difference at *p* < 0.05 by Duncan’s test. Developmental stages S1–S6 correspond to days 30, 50, 80, 90, 100 and 110 after anthesis.

Antioxidant activities were assessed during development by the DPPH (1,1′-diphenyl-2-picrylhydrazyl), FRAP (ferric reducing/antioxidant power), and ABTS^+^ (2,2′-azinobis (3-ethylbenzthiazoline-6-sulfonate) acid) assays in the two jujube cultivars. (Figure 5). The levels of DPPH peaked at S1, and then gradually decreased (a high EC_50_ value indicates low activity) in ‘Tailihong’; levels were higher than in ‘Junzao’ during the ripening period (Figure 5A). Similar change patterns were found for the levels of FRAP and ABTS^+^ (Figure 5B,C). 

Antioxidant activity shows a decreasing trend with time in both cultivars, indicating antioxidant activity at harvest depends on maturity.

To explore the relationship between phenolics and antioxidant activity in jujube skins, the antioxidant capacities of DPPH, FRAP and ABTS were assessed (Table 3). Correlation analyses between TPC, TFC, TFAC, TAC and DPPH, FRAP, ABTS, show that TAC was significantly correlated with antioxidant capacity measured by FRAP radicals (r = 0.889, *p* < 0.01). This indicates anthocyanin has stronger antioxidant activity than the other phenolics, as has been noted previously [34] comparing sensitivities to H_2_O_2_ of anthocyanin and phenolic compounds in apples. Meanwhile, TPC (r = 0.618, *p* < 0.05) and TFAC (r = 0.753, *p* < 0.01) showed closer relationships with scavenging DPPH radical. High positive correlations between antioxidant activity and phenolic compounds suggest these compounds are principally responsible for the antioxidant activity. And the jujube skin extracts had high capacities for scavenging DPPH radicals, ABTS^+^ cation radicals, and FRAP radicals. This may be related to the high contents of total flavanols and total phenolics. The results confirm those of others [11,18,35,36]. 

## 3. Materials and Methods

### 3.1. Plant Materials and Reagents 

Two jujube cultivars, *Ziziphus jujuba* Mill. ‘Tailihong’, and ‘Junzao’, were obtained from the Experimental Station of Jujube of Northwest A&F University in Qingjian, Shaanxi, China. Fruit samples were harvested at six developmental stages on days 30, 50, 80, 90, 100, and 110 after anthesis (DAA). These stages were designated: S1, S2, S3, S4, and S6, respectively. The fruit skins of the two cultivars were removed by hand with a domestic vegetable peeler. The peelings were approximately 2 mm thick. Composite samples were immediately frozen in liquid nitrogen and held at −80 °C pending analysis. At least 15 fruits of each sample and three replicates were performed.

All phenolic standards were purchased from Sigma-Aldrich Co. (St. Louis, MO, USA). *p*-DMACA (*p*-dimethylamino cinnamaldehyde) was obtained from Yuanye Bio-Technology Co. (Shanghai, China). 

### 3.2. Color Phenotypic Measurement 

At least 15 fruits of each cultivar, at each ripening stage, were brought back to the laboratory to determine skin color. Skin color parameters were measured using a CR-400 Minolta Colorimeter (Osaka, Japan) with L*, a*, b* and h^0^ mode, where L*defines lightness (0 = black, 100 = white); a* and b* indicate red-green and blue-yellow, respectively; C* defines saturation and H^0^, hue = arctg (b*/a*), is defined as the hue angle on the color wheel (0° = red/purple, 90° = yellow, 180° = green and 270° = blue) [37]. Skin color was measured on the equator of the fruit. We used the values of a* and h^0^ to represent skin color.

### 3.3. Identification and Quantification of Chlorophylls and Carotenoids

The sample extraction method followed that of Zhao et al. [38] with some modifications. Briefly, samples (approximately 0.15 g) of skin tissue in three biological replicates were extracted with 1.5 mL acetone (containing 0.1 g Butylated hydroxytoluene), mixed well and then placed at 4 °C overnight in darkness. The samples were centrifuged for 15 min at 12,000 rpm and the supernatants were passed through 0.22 μm filters prior to HPLC analysis. 

Chlorophylls and carotenoids were analyzed using liquid chromatography equipment fitted with a diode array detector (1260, Agilent Technology, Palo Alto, CA, USA). Separation was achieved on an Inertsil OD-3 column (5.0 μm, 4.6 × 250 mm; GL Sciences Inc., Tokyo, Japan), preceded by an Inertsil ODS-3 Guard Column (5.0 μm, 4.0 × 10 mm), temperature controlled at 40 °C and with a flow rate of 0.7 mL/min and 10 μL of extract was injected. The solvents were: (A) 90% acetonitrile in water, and (B) ethyl acetate. The elution gradient established was 100% A (0 min), 20% A (14 min), 35% A (20 min), 100% A (30 min). Post-run-time was 10 min. Monitoring was performed at 430 nm for chlorophyll a and chlorophyll b, 450 nm for lutein and β-carotene.

Spectral peaks and retention times of chlorophylls and carotenoids were compared with authentic standards. Quantification was based on peak areas and calibration curves derived from the corresponding authentic standards.

### 3.4. Identification and Quantification of Anthocyanins Extraction 

Samples (about 1.0 g) of fruit skin were extracted with 5 mL of 0.1% HCl in MeOH in an ultrasonic bath for 15 min, mixed well and then placed at 4 °C overnight in the dark and centrifuged next day at 12,000 rpm for 15 min. The supernatants were filtered through a 0.22 μm syringe filter. Three replicates were used for each sample. The extracted compounds were also prepared for analysis of phenolic compounds and antioxidant activity. 

Anthocyanins were analyzed using the same HPLC system and column as the chlorophylls and carotenoids. The HPLC oven controlled at 30 °C with a flow rate of 1.0 mL/min. The solvents were: (A) 10% formic acid (11.36% of 88% formic acid) in water and (B) 10% formic acid (11.36% of 88% formic acid) and 1.36% water in acetonitrile. The elution gradient established was 95% A (5 min), 85% A (25 min), 78% A (42 min), 64%A (60 min) and 95% A (65 min). Post-run-time was 10 min. Monitoring was performed at 520 nm.

Anthocyanins were identified using an LC-ESI-MS/MS system (AB SCIEX LLC, Redwood City, CA, USA) (UPLC, Shim-pack UFLC SHIMADZU CBM20A system; MS, Applied Biosystems 4500 Q TRAP). The analytical conditions followed the method of Wang et al. [29]. Quantification of anthocyanins was as above.

### 3.5. Analyses of Individual Phenolic Compounds

Individual phenolic compounds were analyzed using the same HPLC system and the analytical conditions as the anthocyanin. Monitoring was performed at 280 nm for procyanidin B1, procyanidin B2, procyanidin B3, (−)-epicatechin, (+)-catechin; at 320 nm for gallic acid, chlorogenic acid, caffeic acid, ferulic acid, and cinnamic acid; and at 360 nm for quercetin, quercetin-3-galactoside, quercitrin-3-glucoside, quercetin-3-rutinose, and quercetin-3-rhamnoside. The concentrations of individual phenolic compounds were determined based on peak areas and calibration curves derived from the corresponding authentic phenolic compounds.

### 3.6. Phenolics of Analyses

Total phenolic content (TPC) was determined in triplicate by a modified Folin–Ciocalteu method [39]. Briefly, 2 mL of distilled water, 20 μL of jujube extract and 0.1 mL of Folin–Ciocalteu reagent (1:1 with water) were mixed in a centrifuge tube. After standing for 1 min, 1.00 mL of sodium carbonate (20 g/100 mL) was added. The resultant mixture was then blended and held at room temperature in the dark for 2 h before the absorbance was measured at 765 nm. The TPC was calculated from a calibration curve, using gallic acid as the standard (25–1750 mg/L) to obtain gallic acid equivalents (GAE).

The total flavonoid content (TFC) was determined using a previously reported method [40] with slight modification. Briefly, 225 μL of jujube skin extract was mixed sequentially with 1.98 mL of methanol solution, 150 μL of NaNO_2_ (0.5 M), and 150 μL of AlCl_3_ (0.3 M). After 5 min, 750 μL of NaOH (1 M) was added. The absorbance of the mixture was measured at 510 nm. TFC was calculated from a calibration curve, using rutin as the standard (75–1000 mg/L) to obtain rutin equivalents (RE).

The total flavanol content (TFAC) was measured with p-DMACA [41]. The TFAC was calculated from a calibration curve, using catechin as the standard (6.25–200 mg/L) (catechin equivalents, CE).

The total anthocyanin content (TAC) was measured by the pH differential method [42]. Absorbance was measured at 510 and 700 nm in buffers at pH 1.0 and at 4.5. The TAC is expressed as cyanidin-3-glucoside equivalents. 

### 3.7. Antioxidant Activity of Analyses 

#### 3.7.1. Free-Radical Scavenging of DPPH

The ability to scavenge DPPH radicals was measured by the method of Williams et al. [43], with some modifications. Various conventions of jujube extract (25 μL) were added to 100 μL of a 6 × 10^–5^ M solution of DPPH in methanol. After 30 min incubation period at room temperature, absorbance at 517 nm was measure Trolox was used as standard and results were given as the EC_50_, defined as the concentration of extract (mg/mL) that reduced the DPPH radicals by 50%.

#### 3.7.2. Ferric Reducing Antioxidant Power (FRAP)

The FRAP assay was determined according to the method of Benzie and Strain [44]. The solutions included 300 mM acetate buffer (3.1 g C_2_H_3_NaO_2_·3H_2_O and 16 mL of C_2_H_4_O_2_) at pH 3.6; 10 mM TPTZ solution in 40 mM HCl; and 20 mM FeCl_3_·6H_2_O solution. A fresh working solution was prepared by mixing 25 mL of acetate buffer, 2.5 mL of TPTZ solution, and 2.5 mL of FeCl_3_·6H_2_O solution extract (10 μL in 100 μL of distilled water) was allowed to react with 1.8 mL of the FRAP solution for 10 min at 37 °C. The antioxidant potential was expressed as moles Fe reduced/100 g.

#### 3.7.3. Free-Radical Scavenging of ABTS

The ABTS assay was based on the method of Re et al. [45] with slight modifications. The ABTS^+^ was generated by reacting 7 mM ABTS solution with 2.45 mM potassium persulfate for 12 h in the dark at room temperature. The ABTS^+^ reagent was diluted with ethanol to achieve an absorbance of 0.70 ± 0.02 at 734 nm. After 25 μL of either sample or Trolox standard were added to 100 μL of the diluted ABTS^+^ reagent, absorbance at 734 nm was measured at exactly 1 min. Trolox was used as standard and the results were given as the EC50, defined as the concentration of extract (mg/mL) that reduced the ABTS radicals by 50%.

### 3.8. Statistical Analyses

Values are expressed as means ± standard deviations (SD) from three replicates. All data analyses were carried out using SPSS 23.0 (SPSS, IBM Corporation, Armonk, NY, USA). One-way analysis of variance (ANOVA) and Duncan’s multiple range tests were used to identify significance of difference. Differences were considered to be significant at *p* < 0.05. Two-tailed Pearson’s correlation coefficients were calculated to describe associations between phenolics level and antioxidant activity. The data were plotted using SigmaPlot 12.0 software (SigmaPlot, SYSTAT Corporation, San Jose, CA, USA).

## 4. Conclusions 

These results indicate that fruit skin color at maturity is due to the levels of flavonoids, carotenoids, and anthocyanins. Cyanidin-3-glucoside, cyanidin-3-rutinoside, cyanidin and delphinidin-3-glucoside were identified through HPLC retention times, LC-MS/MS and PhotoDiode Arraray (PDA). TPC, TFC and TFAC first increased and then decreased sharply in both cultivars, while TAC decreased with fruit development. Meanwhile, Antioxidant activity measured by DPPH, FRAP and ABTS^+^ reduced gradually during fruit development in both cultivars. The levels of phenolics vary with maturity. Overall, this study comprehensive analyzed the pigment formation during fruit development in Chinese jujube and the changes in phenolic compounds and antioxidant activity. The present study contributes to the appreciation of the nutritional and functional value of jujube fruit.

## Figures and Tables

**Table 1:** Contents of individual phenolics in the skins of ‘Tailihong’ and ‘Junzao’ jujube fruit at different development stages.

Developmental Stage	Cultivar	Flavanol Component (mg/kg FW)						Phenolic Acids Component (mg/kg FW)				
		Procyanidin B1	Procyanidin B2	Procyanidin B3	(−)-Epicatechin	(+)-Catechin	Quercetin	Gallic Acid	Chlorogenic Acid	Caffeic Acid	Ferulic Acid	Cinnamic Acid
S1	Tailihong	107.52 ± 4.33 ^c^	374.16 ± 31.47 ^c^	237.35 ± 15.6 ^c^	27.94 ± 2.57 ^a^	58.19 ± 3.62 ^bc^	16.19 ± 1.19 ^g^	61.22 ± 2.8 ^fg^	94.75 ± 3.25 ^a^	22.65 ± 0.66 ^a^	14.98 ± 1.45 ^b^	44.64 ± 1.34 ^a^
	Junzao	80.51 ± 7.38 ^d^	300.67 ± 21.94 ^d^	112.03 ± 5.04 ^d^	20.23 ± 0.8 ^b^	33.42 ± 1.05 ^de^	26.21 ± 1.35 ^cd^	77.37 ± 3.73 ^de^	73.67 ± 4.77 ^b^	15.45 ± 0.13 ^d^	3.31 ± 0.2 ^d^	18.29 ± 0.35 ^cd^
S2	Tailihong	100.26 ± 4.45 ^cd^	391.15 ± 15.44 ^c^	341.56 ± 7.81 ^b^	10.91 ± 0.25 ^d^	56 ± 4.18 ^bc^	22.12 ± 0.24 ^de^	119 ± 1.33 ^b^	61.29 ± 3.09 ^c^	16.69 ± 0.21 ^cd^	50 ± 2.66 ^a^	36.5 ± 2.84 ^a^
	Junzao	31.19 ± 0.66 ^e^	491.29 ± 11.38 ^b^	128.97 ± 0.69 ^d^	15.57 ± 0.77 ^c^	73.36 ± 0.07 ^ab^	23.53 ± 0.83 ^de^	69.54 ± 1.67 ^ef^	46.86 ± 3.71 ^d^	20.22 ± 1.21 ^b^	2.77 ± 0.01 ^d^	40.24 ± 3.2 ^a^
S3	Tailihong	286.06 ± 15.65 ^a^	888.18 ± 15.94 ^a^	476.48 ± 15.65 ^a^	8.16 ± 0.73 ^e^	82.08 ± 6.49 ^a^	28.33 ± 1 ^c^	157.01 ± 6.58 ^a^	48.96 ± 2.66 ^d^	17.67 ± 1.17 ^c^	16.84 ± 1.25 ^b^	42.41 ± 1.04 ^a^
	Junzao	31.97 ± 0.28 ^e^	228.8 ± 8.3 ^e^	38.88 ± 3.36 ^e^	12.98 ± 0.84 ^d^	21.78 ± 0.02 ^e^	42.79 ± 0.7 ^a^	74.39 ± 0.22 ^de^	57.4 ± 0.62 ^c^	7.42 ± 0.6 ^fg^	7.91 ± 0.08 ^c^	8.56 ± 0.67 ^e^
S4	Tailihong	187.63 ± 29.07 ^b^	417.54 ± 63.91 ^c^	248.42 ± 9.99 ^c^	7.29 ± 0.38 ^ef^	45.37 ± 4.24 ^cd^	16.62 ± 1.96 ^g^	83.87 ± 9.86 ^d^	44.57 ± 5.15 ^d^	13.3 ± 0.56 ^e^	14.15 ± 0.7 ^b^	28.5 ± 1.03 ^b^
	Junzao	290.3 ± 0.82 ^a^	200.07 ± 0.53 ^ef^	491.94 ± 36.18 ^a^	11.39 ± 0.07 ^d^	20 ± 1.69 ^e^	24.87 ± 0.39 ^cde^	69.64 ± 2.28 ^ef^	19.43 ± 0.01 ^f^	ND	3.01 ± 0.08 ^d^	27.31 ± 1.99 ^b^
S5	Tailihong	47.43 ± 6.1 ^e^	406.89 ± 15.52 ^c^	44.51 ± 4.92 ^e^	5.46 ± 0.23 ^f^	27.25 ± 0.66 ^de^	21.48 ± 1.32 ^ef^	75.2 ± 4.49 ^de^	21.19 ± 4.46 ^f^	5.34 ± 1.13 ^h^	4.38 ± 0.27 ^d^	12.24 ± 0.13 ^de^
	Junzao	25.71 ± 1.14 ^e^	85.37 ± 3.2 ^h^	52.16 ± 10.73 ^e^	7.57 ± 0.59 ^ef^	18.69 ± 0.6 ^e^	22.51 ± 0.94 ^de^	93.8 ± 5.2 ^c^	31.17 ± 1.18 ^e^	6.14 ± 0.45 ^gh^	3.54 ± 0.26 ^d^	12.54 ± 0.97 ^de^
S6	Tailihong	76.94 ± 4.58 ^d^	129.38 ± 2.16 ^gh^	66.07 ± 5.98 ^e^	7.89 ± 0.64 ^e^	32.34 ± 2.25 ^de^	17.59 ± 1.46 ^fg^	55.34 ± 4.51 ^g^	42.54 ± 4.92 ^d^	8.53 ± 1.28 ^f^	7.46 ± 0.53 ^c^	24.04 ± 1.51 ^bc^
	Junzao	49.12 ± 7.7 ^e^	153.26 ± 12.32 ^fg^	48.63 ± 7.25 ^e^	8.32 ± 0.02 ^e^	16.38 ± 0.24 ^e^	32.56 ± 7.8 ^b^	63.96 ± 4.41 ^fg^	47.24 ± 0.13 ^d^	7.56 ± 0.3 ^fg^	2.89 ± 0.04 ^d^	14.65 ± 1.06 ^de^

Values of three replicates are expressed as the means ± SD. Different letters (a–h) in the same column indicate significant different at *p* < 0.05 by Duncan’s test. (ND, not detected). Developmental stages S1–S6 correspond to days 30, 50, 80, 90, 100, and 110 after anthesis.

**Table 2:** Contents of individual phenolics in the skins of ‘Tailihong’ and ‘Junzao’ jujube fruit at different developmental stages.

Developmental Stage	Cultivar	Flavonol Component (mg/kg FW)			
		Quercetin-3-galactoside	Quercitrin-3-glucoside	Quercetin-3-rutinose	Quercetin-3-rhamnoside
S1	Tailihong	37.53 ± 2.75 ^bc^	29.27 ± 2.43 ^e^	1.39 ± 0.11 ^g^	30.2 ± 1.61 ^ef^
	Junzao	81.36 ± 6.95 ^a^	324.31 ± 19.51 ^a^	8.37 ± 0.61 ^a^	244.9 ± 11.62 ^a^
S2	Tailihong	19.88 ± 1.22 ^def^	105.08 ± 2.07 ^c^	2.1 ± 0.16 ^f^	51.25 ± 0.34 ^de^
	Junzao	34.24 ± 0.25 ^c^	161.72 ± 19.79 ^b^	4.61 ± 0.26 ^c^	151.03 ± 3.02 ^b^
S3	Tailihong	27.09 ± 0.75 ^d^	155.1 ± 7.47 ^b^	2.64 ± 0.19 ^ef^	69.89 ± 2.16 ^cd^
	Junzao	25.04 ± 2.23 ^de^	35.37 ± 4.41 ^e^	3.02 ± 0.44 ^e^	87.17 ± 5.33 ^c^
S4	Tailihong	19.49 ± 1.66 ^ef^	76.8 ± 5.01 ^d^	2.81 ± 0.34 ^e^	39.4 ± 3.93 ^ef^
	Junzao	42.87 ± 2.88 ^b^	97.1 ± 2.61 ^c^	5.86 ± 0.13 ^b^	155.8 ± 1.01 ^b^
S5	Tailihong	13.73 ± 1.01 ^fg^	64.94 ± 17.7 ^d^	3.8 ± 0.45 ^d^	48.36 ± 2.07 ^e^
	Junzao	5.7 ± 0.8 ^hi^	7.67 ± 0.19 ^f^	1.19 ± 0.18 ^g^	30.08 ± 2.68 ^ef^
S6	Tailihong	12.13 ± 0.97 ^gh^	9.5 ± 2.83 ^f^	3.08 ± 0.27 ^e^	24.79 ± 1.04 ^f^
	Junzao	3.52 ± 0.29 ^i^	40.94 ± 2.16 ^e^	1.21 ± 0.1 ^g^	166.27 ± 1.23 ^b^

Values of three replicates are expressed as the means ± SD. Different letters (a–i) in the same column indicate significant different at *p* < 0.05 by Duncan’s test. Developmental stages S1–S6 correspond to days 30, 50, 80, 90, 100 and 110 after anthesis.

**Table 3:** Pearson’s correlation coefficients of phenolics (TPC, TFC, TFAC, and TAC) and antioxidant capacity (via DPPH, FRAP and ABTS^+^) in the skins of ‘Tailihong’ and ‘Junzao’ jujube fruit.

	DPPH	FRAP	ABTS^+^
TPC	−0.618 *	0.591 *	−0.585 *
TFC	−0.615 *	0.515	−0.401
TFAC	−0.753 **	0.789 **	−0.653 *
TAC	−0.714 **	0.889 **	−0.730 **

** *p* < 0.01; * *p* < 0.05.

## Supplementary Materials

The following are available online, Figure S1. Anthocyanin component analysis in the skins of jujube fruit cultivars ‘Tailihong’ and ‘Junzao’ (A) HPLC chromatograms of anthocyanin profiles of two jujube cultivars. The MS2 spectra of (B) cyanidin 3-glucoside, (C) delphinidin-3-glucoside, (D) cyanidin 3-rutinoside and (E) cyanidin. (F-I) UV spectra of peak A1–A4. Figure S2. HPLC chromatograms of all classes of compounds jujube fruit skins. Table S1. Pearson’s correlation coefficients of phenolics (TPC, TFC, TFAC, and TAC) and color difference (CIE a* and h*) in the skins of ‘Junzao’. Table S2. Pearson’s correlation coefficients of phenolics (TPC, TFC, TFAC and TAC) and color difference (CIE a* and h*) in the skins of ‘Tailihong’.

## References

[1] Huang J., Zhang C.M., Zhao X., Fei Z.J., Wan K.K., Zhang Z., Pang X.M., Yin X., Bai Y., Sun X.Q. (2016). The Jujube Genome Provides Insights into Genome Evolution and the Domestication of Sweetness/Acidity Taste in Fruit Trees. PLoS Genet..

[2] Zhang J.L., Li C.L. (2017). Forestry Statistical Yearbook 2016.

[3] Pahuja M., Mehla J., Reeta K.H., Joshi S., Gupta Y.K. (2011). Hydroalcoholic extract of *Zizyphus jujuba* ameliorates seizures, oxidative stress, and cognitive impairment in experimental models of epilepsy in rats. Epilepsy Behav..

[4] Plastina P., Bonofiglio D., Vizza D., Fazio A., Rovito D., Giordano C., Barone I., Catalano S., Gabriele B. (2012). Identification of bioactive constituents of *Ziziphus jujube* fruit extracts exerting antiproliferative and apoptotic effects in human breast cancer cells. J. Ethnopharmacol..

[5] Yeung W.F., Chung K.F., Poon M.M., Ho F.Y., Zhang S.P., Zhang Z.J., Ziea E.T., Wong V.T. (2012). Chinese herbal medicine for insomnia: A systematic review of randomized controlled trials. Sleep Med. Rev..

[6] Chen J., Li Z., Maiwulanjiang M., Zhang W.L., Zhan J.Y., Lam C.T., Zhu K.Y., Yao P., Choi R.C., Lau D.T. (2013). Chemical and biological assessment of *Ziziphus jujuba* fruits from China: Different geographical sources and developmental stages. J. Agric. Food Chem..

[7] Du L.J., Gao Q.H., Ji X.L., Ma Y.J., Xu F.Y., Wang M. (2013). Comparison of flavonoids, phenolic acids, and antioxidant activity of explosion-puffed and sun-dried jujubes (*Ziziphus jujuba* Mill.). J. Agric. Food Chem..

[8] Kim K., Tsao R., Yang R., Cui S. (2006). Phenolic acid profiles and antioxidant activities of wheat bran extracts and the effect of hydrolysis conditions. Food Chem..

[9] Rodriguez-Mateos A., Vauzour D., Krueger C.G., Shanmuganayagam D., Reed J., Calani L., Mena P., Del Rio D., Crozier A. (2014). Bioavailability, bioactivity and impact on health of dietary flavonoids and related compounds: An update. Arch. Toxicol..

[10] Chen C.S., Zhang D., Wang Y.Q., Li P.M., Ma F.W. (2012). Effects of fruit bagging on the contents of phenolic compounds in the peel and flesh of ‘Golden Delicious’, ‘Red Delicious’, and ‘Royal Gala’ apples. Sci. Hortic..

[11] Wang B., Huang Q., Venkitasamy C., Chai H., Gao H., Cheng N., Cao W., Lv X., Pan Z. (2016). Changes in phenolic compounds and their antioxidant capacities in jujube (*Ziziphus jujuba* Miller.) during three edible maturity stages. LWT Food Sci. Technol..

[12] Wu C.S., Gao Q.H., Guo X.D., Yu J.G., Wang M. (2012). Effect of ripening stage on physicochemical properties and antioxidant profiles of a promising table fruit ‘pear-jujube’ (*Zizyphus jujuba* Mill.). Sci. Hortic..

[13] Canals R., Llaudy M.C., Valls J., Canals J.M., Zamora F. (2005). Influence of ethanol concentration on the extraction of color and phenolic compounds from the skin and seeds of tempranillo grapes at different stages of ripening. J. Agric. Food Chem..

[14] Da Silva F.L., Escribano-Bailon M.T., Alonso J.J.P., Rivas-Gonzalo J.C., Santos-Buelga C. (2007). Anthocyanin pigments in strawberry. LWT Food Sci. Technol..

[15] Rivera-Lopez J., Ordorica-Falomir C., Wesche-Ebeling P. (1999). Changes in anthocyanin concentration in Lychee (*Litchi chinensis* Sonn.) pericarp during maturation. Food Chem..

[16] Kevresan Z.S., Mastilovic J.S., Mandic A.I., Torbica A.M. (2013). Effect of Different Ripening Conditions on Pigments of Pepper for Paprika Production at Green Stage of Maturity. J. Agric. Food Chem..

[17] Perrin F., Brahem M., Dubois-Laurent C., Huet S., Jourdan M., Geoffriau E., Peltier D., Gagne S. (2016). Differential Pigment Accumulation in Carrot Leaves and Roots during Two Growing Periods. J. Agric. Food Chem..

[18] Xie P.J., You F., Huang L.X., Zhang C.H. (2017). Comprehensive assessment of phenolic compounds and antioxidant performance in the developmental process of jujube (*Ziziphus jujuba* Mill.). J. Funct. Foods.

[19] Zhang H., Jiang L., Ye S., Ye Y., Ren F. (2010). Systematic evaluation of antioxidant capacities of the ethanolic extract of different tissues of jujube (*Ziziphus jujuba* Mill.) from China. Food Chem. Toxicol..

[20] Castrejón A.D.R., Eichholz I., Rohn S., Kroh L.W., Huyskens-Keil S. (2008). Phenolic profile and antioxidant activity of highbush blueberry (*Vaccinium corymbosum* L.) during fruit maturation and ripening. Food Chem..

[21] Almansa S., Hernandez F., Legua P., Nicolas-Almansa M., Amoros A. (2016). Physico-chemical and physiological changes during fruit development and on-tree ripening of two Spanish jujube cultivars (*Ziziphus jujuba* Mill.). J. Sci. Food Agric..

[22] Lancaster J.E., Lister C.E., Reay P.F., Triggs C.M. (1997). Influence of pigment composition on skin color in a wide range of fruit and vegetables. J. Am. Soc. Hortic. Sci..

[23] Alniami J.H., Saggar R.A.M., Abbas M.F. (1992). The physiology of ripening of jujube (*Ziziphus jujuba* Mill.). Sci. Hortic..

[24] Bastos V.J., Neves L.C., da Silva P.M.C., Shahab M., Colombo R.C., Roberto S.R. (2016). Harvest point determination of indian jujube fruit (*Ziziphus mauritiana* L.) based on physicochemical and functional parameters. Sci. Hortic..

[25] Hertog M.G.L., Kromhout D., Aravanis C., Blackburn H., Buzina R., Fidanza F., Giampaoli S., Jansen A., Menotti A., Nedeljkovic S. (1995). Flavonid intake and long-term risk of coronary-heart-disease and cancer in the 7 countries study. Arch. Intern. Med..

[26] Choi S.H., Ahn J.B., Kim H.J., Im N.K., Kozukue N., Levin C.E., Friedman M. (2012). Changes in free amino acid, protein, and flavonoid content in jujube (*Ziziphus jujuba*) fruit during eight stages of growth and antioxidative and cancer cell inhibitory effects by extracts. J. Agric. Food Chem..

[27] Pu Y., Ding T., Zhang N., Jiang P., Liu D. (2017). Identification of bitter compounds from dried fruit of *Ziziphus jujuba* cv. Junzao. Int. J. Food Prop..

[28] Bai L., Zhang H., Liu Q.C., Zhao Y., Cui X.Q., Guo S., Zhang L., Ho C.T., Bai N.S. (2016). Chemical characterization of the main bioactive constituents from fruits of *Ziziphus jujuba*. Food Funct..

[29] Wang Z., Cui Y., Vainstein A., Chen S., Ma H. (2017). Regulation of Fig (*Ficus carica* L.) Fruit Color: Metabolomic and Transcriptomic Analyses of the Flavonoid Biosynthetic Pathway. Front. Plant Sci..

[30] Gao Q.H., Wu C.S., Wang M., Xu B.N., Du L.J. (2012). Effect of drying of jujubes (*Ziziphus jujuba* Mill.) on the contents of sugars, organic acids, alpha-tocopherol, beta-carotene, and phenolic compounds. J. Agric. Food Chem..

[31] Zozio S., Servent A., Cazal G., Mbeguie A.M.D., Ravion S., Pallet D., Abel H. (2014). Changes in antioxidant activity during the ripening of jujube (*Ziziphus mauritiana* Lamk.). Food Chem..

[32] Carlsen M.H., Halvorsen B.L., Holte K., Bohn S.K., Dragland S., Sampson L., Willey C., Senoo H., Umezono Y., Sanada C. (2010). The total antioxidant content of more than 3100 foods, beverages, spices, herbs and supplements used worldwide. Nutr. J..

[33] You F., Huang L.X., Zhang C.H., Xie P.J., Zhang Y.L. (2014). Changes in phenolic content and DPPH radical scavengingactivity during the development in the skin of ‘Dongzao’ jujube. Food Sci. Biotechnol..

[34] Bi X., Zhang J., Chen C., Zhang D., Li P., Ma F. (2014). Anthocyanin contributes more to hydrogen peroxide scavenging than other phenolics in apple peel. Food Chem..

[35] Choi S.H., Ahn J.B., Kozukue N., Levin C.E., Friedman M. (2011). Distribution of free amino acids, flavonoids, total phenolics, and antioxidative activities of Jujube (*Ziziphus jujuba*) fruits and seeds harvested from plants grown in Korea. J. Agric. Food Chem..

[36] Kou X., Chen Q., Li X., Li M., Kan C., Chen B., Zhang Y., Xue Z. (2015). Quantitative assessment of bioactive compounds and the antioxidant activity of 15 jujube cultivars. Food Chem..

[37] McGuire R.G. (1992). Repoting of objective color measurements. Hortscience.

[38] Zhao X.Q., Yuan Z.H., Yin Y.L., Feng L.J., Yuan Z., Wilkins E., Wang D. (2015). Patterns of Pigment Changes in Pomegranate (*Punica granatum* L.) Peel during Fruit Ripening. Proceedings of the III International Symposium on Pomegranate and Minor Mediterranean Fruits.

[39] Wang H., Chen F., Yang H., Chen Y., Zhang L., An H. (2012). Effects of ripening stage and cultivar on physicochemical properties and pectin nanostructures of jujubes. Carbohydr. Polym..

[40] Jia Z., Tang M.C., Wu J.M. (1999). The determination of flavonoid contents in mulberry and their scavenging effects on superoxide radicals. Food Chem..

[41] Li Y.G., Tanner G., Larkin P. (1996). The DMACA-HCl protocol and the threshold proanthocyanidin content for bloat safety in forage legumes. J. Sci. Food Agric..

[42] Giusti M.M., Wrolstad R.E. (2001). Characterization and measurement of Anthocyanins by UV-Visible Spectroscopy. Curr. Protoc. Food Anal. Chem..

[43] Brand-Williams W., Cuvelier M.E., Berset C. (1995). Use of a free-radical method to evaluate antioxidant activity. LWT Food Sci. Technol..

[44] Benzie I.F.F., Strain J.J. (1996). The ferric reducing ability of plasma (FRAP) as a measure of “antioxidant power”: The FRAP assay. Anal. Biochem..

[45] Re R., Pellegrini N., Proteggente A., Pannala A., Yang M., Rice-Evans C. (1999). Antioxidant activity applying an improved ABTS radical cation decolorization assay. Free Radic. Biol. Med..

