# Supplementary material for: Comparative Analysis of Pigments, Phenolics, and Antioxidant Activity of Chinese Jujube (Ziziphus jujuba Mill.) during Fruit Development
Source: Molecules. 2018 Aug 1;23(8):1917. doi: 10.3390/molecules23081917 (PMC6222768; doi:10.3390/molecules23081917)
Supplement: Supplementary file 1 [file molecules-23-01917-s001.pdf]

Identification of anthocyanins

Four anthocyanins were identified in these two cultivars (Figure S1). The identification of anthocyanins was through HPLC retention times, LC-MS/MS and PhotoDiode Arraray(PDA). The HPLC peaks A1, A2, A4 and A3 correspond to the retention times of cyanidin-3 glucoside, cyanidin-3-rutinoside, cyanidin and delphenidin-3-glucoside (Figure S1 A). The PDA data shows these peaks were indeed anthocyanin peaks (Figure S1 F-I). The MS data show that the peak A1 produced molecular mass ($[M]^+$ *m/z* 449, which was further fragmented into MS/MS *m/z* 287 and 192) (Figure S1 B), matching cyanidin 3-glucoside. The minority peak A3 was interpreted as delphinidin-3-glucoside, based on the mass spectral characteristics ($[M]^+$ *m/z* 464, MS/MS *m/z* 303) (Figure S1 C). The MS data of Peak A2 ($[M]^+$ *m/z* 595, MS/MS *m/z* 449 and 287) (Figure S1 D) was observed, which indicates this anthocyanin contained one cyanidin, one deoxyhexosyl and one hexosyl. This anthocyanin was tentatively identified as cyanidin-3-rutinoside, and is here reported in jujubes for the first time. Based on the molecular weight of 287($[M]^+$), peak A4 was tentatively identified as cyanidin (Figure S1 E).

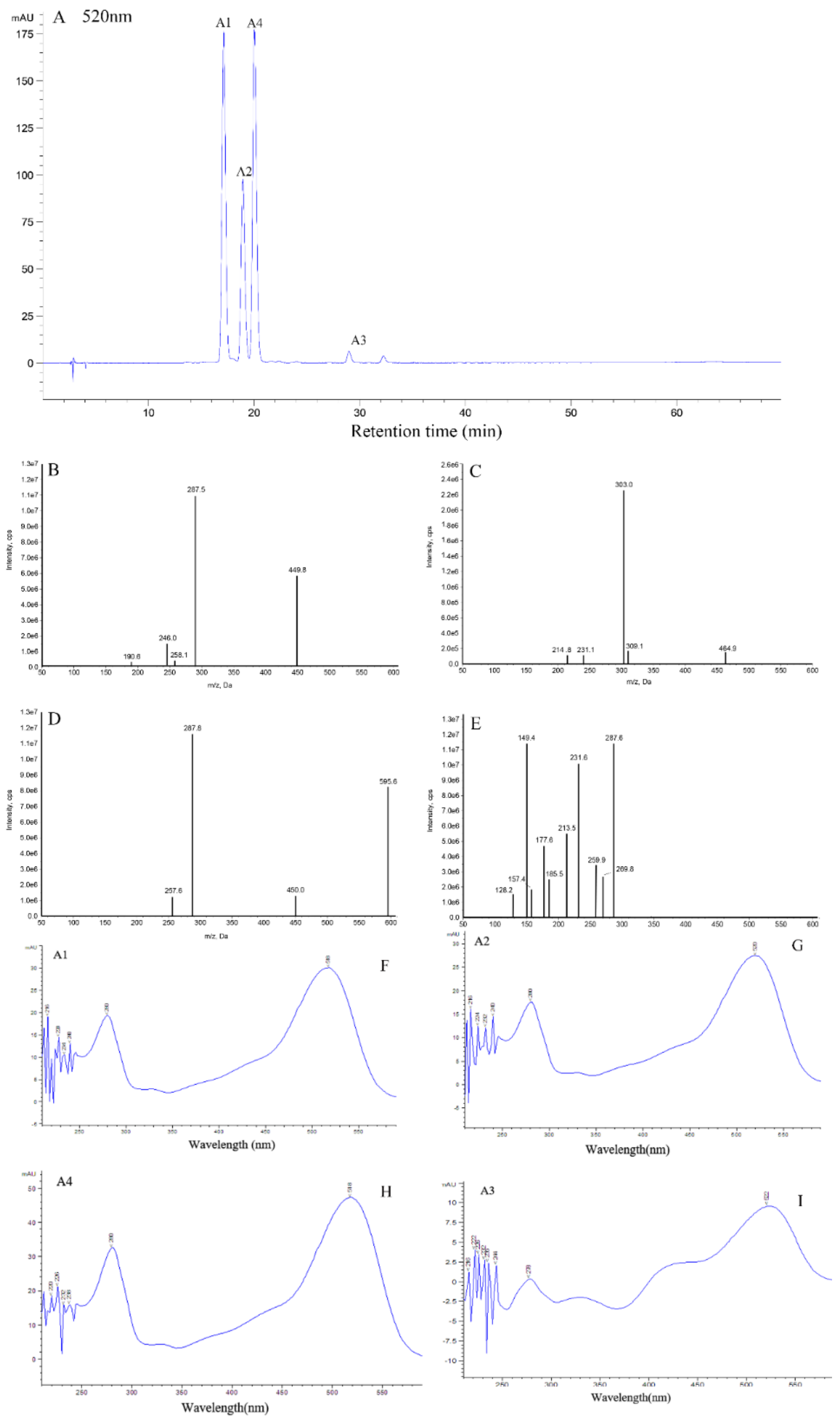

Figure S1. Anthocyanin component analysis in the skins of the jujube fruit. (A) HPLC chromatograms of anthocyanin profiles of jujube fruit skins. The MS2 spectra of (B) cyanidin 3-glucoside, (C) delphinidin-3-glucoside, (D) cyanidin 3-rutinoside and (E) cyanidin. (F-I) UV spectra of peak A1- A4.

Table S1. Pearson's correlation coefficients of phenolics (TPC, TFC, TFAC and TAC) and color difference (CIE a* and h*) in the skins of 'Junzao'.

	TPC	TFC	TFAC	TAC
a*	-0.840*	-0.801	-0.879*	-0.042
h*	0.900*	0.827*	0.827*	-0.062

*** p<0.01; *p<0.05*

Table S2. Pearson's correlation coefficients of phenolics (TPC, TFC, TFAC and TAC) and color difference (CIE a* and h*) in the skins of 'Tailihong'.

	TPC	TFC	TFAC	TAC
a*	-0.183	-0.044	0.363	0.465
h*	-0.086	0.292	-0.201	-0.643

*** p<0.01; *p<0.05*

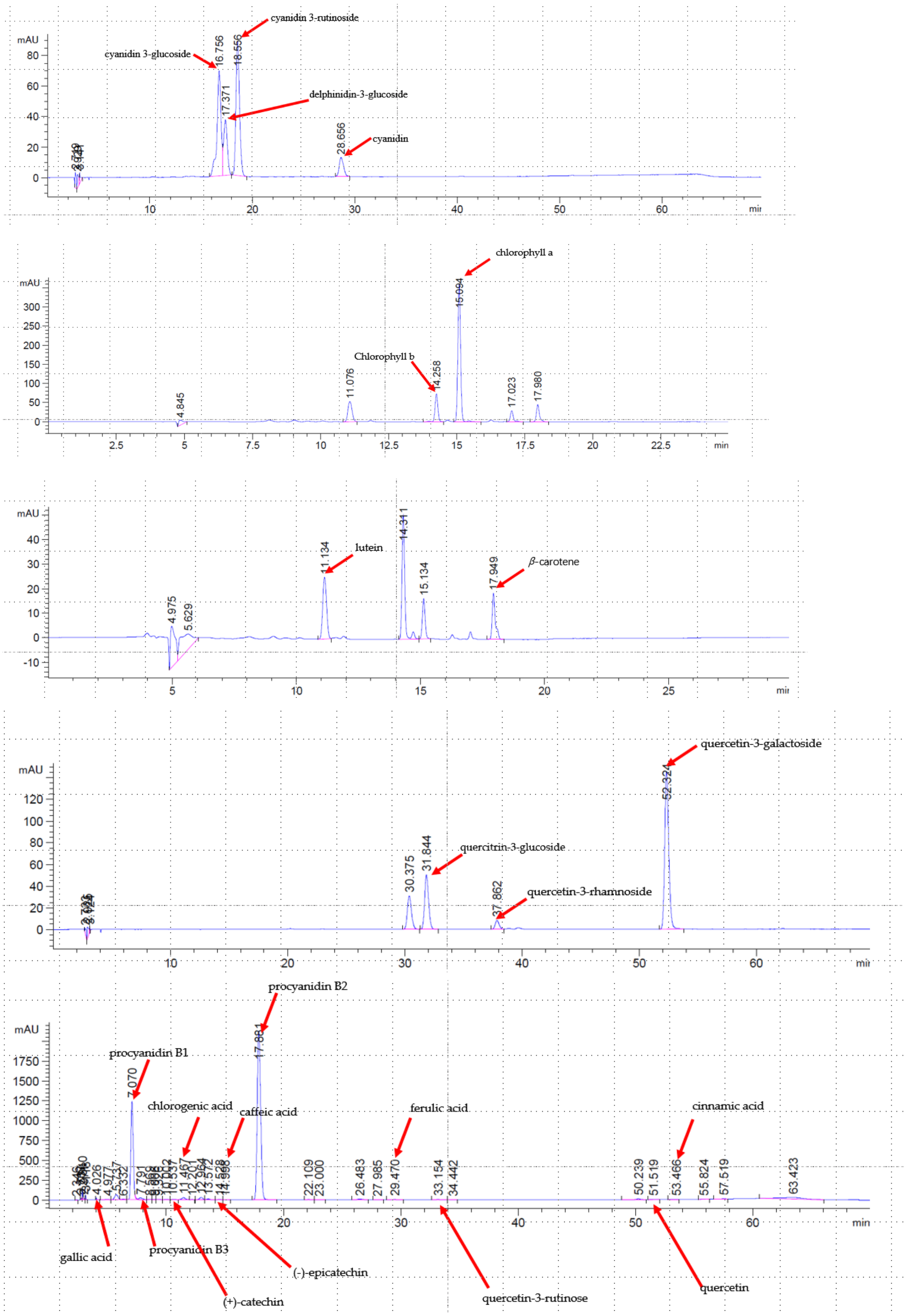

Figure S2. HPLC chromatograms of all classes of compounds jujube fruit skins.
